# *Neospora caninum* surface antigen 1 is a major determinant of the pathogenesis of neosporosis in nonpregnant and pregnant mice

**DOI:** 10.3389/fmicb.2023.1334447

**Published:** 2024-01-08

**Authors:** Hanan H. Abdelbaky, Md. Masudur Rahman, Naomi Shimoda, Yu Chen, Tanjila Hasan, Nanako Ushio, Yoshifumi Nishikawa

**Affiliations:** ^1^National Research Center for Protozoan Diseases, Obihiro University of Agriculture and Veterinary Medicine, Inada-cho, Obihiro, Hokkaido, Japan; ^2^Department of Pathology, Faculty of Veterinary, Animal and Biomedical Sciences, Sylhet Agricultural University, Sylhet, Bangladesh; ^3^Department of Medicine and Surgery, Faculty of Veterinary Medicine, Chattogram Veterinary and Animal Sciences University, Khulshi, Chattogram, Bangladesh

**Keywords:** Neospora caninum, NcSAG1, CRISPR/Cas9, gene editing, pregnancy, vertical transmission, Balb/c mouse

## Abstract

**Introduction:**

NcSAG1 is one of most widely investigated antigens of *Neospora caninum* in various research fields. Such studies demonstrated the proficiency of NcSAG1 in the regulatory process of parasite adhesion and invasion of host cells. Accordingly, the contribution of NcSAG1 to the pathogenesis of neosporosis can undoubtedly be extrapolated, but direct evidence is lacking. Herein, we provide the first successful attempt at the gene disruption of NcSAG1 and novel data on the invasion and virulence potentials of *N. caninum in vitro* and *in vivo*.

**Methods:**

The disruption of the NcSAG1 gene was applied using the CRISPR/Cas9 system and confirmed by PCR, western blot and indirect fluorescent antibody tests as NcSAG1 knockout parasites (NcSAG1KO). Then, we investigated the role of NcSAG1 in the growth kinetics of the parasite *in vitro*.

**Results and discussion:**

The deletion of the NcSAG1 gene significantly decreased the infection rate and reduced the egress rate of the parasite. An *in vivo* study using nonpregnant female and male BALB/c mice revealed a significantly higher survival rate and lower body weight change in the group infected with the NcSAG1KO parasite than in the parental strain (Nc-1)-infected group. Regarding the vertical transmission model of BALB/c mice, the absence of the NcSAG1 gene significantly enhanced the survival of pups and greatly lowered the parasite burden in the brains of pups. In conclusion, our study suggested NcSAG1 as a key molecule in the pathogenesis of *N. caninum*.

## Introduction

*Neospora caninum*, which is a protozoan parasite closely related to *Toxoplasma gondii*, can infect a wide range of intermediate hosts. *In vitro*, *N. caninum* has the capacity to be maintained in a wide range of cell cultures. However, in contrast to *T. gondii*, *N. caninum* is a primary causative agent of abortion or fetal abnormalities in cattle and, to a lesser extent, in sheep and other ruminants (Goodswen et al., 2013). Three developmental and infective stages have been reported for *N. caninum*: tachyzoite (rapidly growing stage), bradyzoite (slowly growing stage) and sporozoite (existing in oocysts). The sexual cycle of *N. caninum* occurs in dogs and some other canids that act as definitive hosts that are affected by neurological conditions, while many other animals, including cattle, act as intermediate hosts that harbor only the asexual stage (Dubey and Schares, 2011; Fereig and Nishikawa, 2020). Although cellular immune responses are needed for controlling *N. caninum*, both innate and acquired immunity are effective against infection. *N. caninum* can efficiently manipulate several subsets of immune cells, including macrophages and T lymphocytes. Several previous studies revealed the critical role of IFN-γ in the direct killing of the parasite. These studies have been confirmed using different animal models, cell types and experimental approaches (reviewed by Aguado-Martinez et al., 2017; Nishikawa, 2017; Fereig and Nishikawa, 2020). These *N. caninum*-host interactions are regulated by several key factors produced from the parasite. However, no vaccine or effective drugs are currently available for the prevention of bovine or canine neosporosis. Thus, control is primarily based on the detection and culling of infected animals. The application of hygienic measures at the farm level and quarantine measures for imported animals can minimize the economic losses due to neosporosis (Ortega-Mora et al., 2007; Sinnott et al., 2017).

Vertical transmission is the main route of infection of *N. caninum* rather than horizontal rout via oocysts (Dubey and Schares, 2006). Vertical transmission of *N. caninum* and its transplacental transmission results in the persistent transfer of the parasites from infected dams to their fetuses, leading to the wide distribution of *N. caninum* in the field (Dubey et al., 2006). Generally, type 1 and type 2 immune responses are correlated with both vertical transmission and abortion caused by *N. caninum* infection (Quinn et al., 2002). However, abortion induced by *N. caninum* infection has not been observed in the murine model. To understand pathological mechanism of *N. caninum* infection during gestation, vertical transmission models using BALB/c mice (Nishikawa et al., 2001; Omata et al., 2004) and C57BL/6 mice (Ramamoorthy et al., 2007a 2007b; Abdou et al., 2022) were investigated. The reduction in the transplacental transmission was involved in lower levels of IL-4 and higher levels of IFN-γ production (Long and Baszler, 2000), indicating type 1 immune response can control the vertical transmission of *N. caninum*. Therefore, balance of type 1 and type 2 immune responses will be associated with protection and vertical transmission caused by *N. caninum* infection. Recently, role of *N. caninum* molecule on the vertical transmission has been focused. In fact, dense granule protein 7 (NcGRA7), or rhoptry protein NcROP40-deficient parasites reduced vertical transmission in mice (Abdou et al., 2022; Rico-San Román et al., 2022).

Surface antigens of *N. caninum* tachyzoites regulate the process of adhesion and invasion of host cells. Two major surface antigens, NcSAG1 and NcSRS2, were identified and widely studied (Hemphill and Gottstein, 1996). NcSAG1 is anchored on the surface of tachyzoites by glycosylphosphatidylinositol (GPI) anchors (Howe et al., 1998), and NcSRS2 is also anchored on GPI (Nishikawa et al., 2002). Both antigens were observed to be downregulated during the tachyzoite to bradyzoite stage (Hemphill and Gottstein, 1996). NcSAG1 is one of the most studied *N. caninum*-derived molecules in numerous studies of different purposes. It is extensively used as a potent antigen for *N. caninum* epidemiologic studies, diagnostic systems, and vaccine studies (Sinnott et al., 2017; Fereig and Nishikawa, 2020). These studies revealed the potential of NcSAG1 in stimulating cellular or humoral immune responses. Our previous study showed that a monoclonal antibody against NcSAG1 inhibited the invasion of *N. caninum* into host cells (Nishikawa et al., 2000), suggesting the physiological function of this antigen in parasite biology. However, the pivotal role of NcSAG1 in the pathogenesis of neosporosis is still unknown.

Although numerous approaches have been used to study gene function, knocking out such genes is considered the most efficient and reliable method. Recently, the CRISPR/Cas9 system has been successfully developed and evaluated against numerous *N. caninum* dense granule genes (NcGRA6, NcGRA7, NcGRA14 and NcCyp) and has led to multiple novel interesting datasets (Nishikawa et al., 2018). As expected, the role of NcGRA7 was superior to that of the other abovementioned genes, as evidenced by its contribution to parasite virulence associated with a higher inflammatory response and parasite burden in infected mice with Nc-1 compared with that with NcGRA7-deficient parasites (Nishikawa et al., 2018). Thus, to investigate the role of NcSAG1, a gene editing system based on CRISPR/Cas9 was used in this study. Herein, the deletion of the NcSAG1 gene restricted the invasion and egress of the parasites *in vitro*. Moreover, our study demonstrated NcSAG1 as a determinant molecule for virulence of *N. caninum* either in nonpregnant or pregnant mouse models. The successful elimination of NcSAG1 stands out, marking an important milestone in *Neospora* research.

## Materials and methods

### Ethics statement

All experimental works were performed in the current study, according to recommendations and procedures specified by the Guide for the Care and Use of Laboratory Animals of the Ministry of Education, Culture, Sports, Science and Technology, Japan. The methods were approved by the Committee on the Ethics of Animal Experiments at the Obihiro University of Agriculture and Veterinary Medicine (permission numbers 22–96, 21–30, 20–23, 19–51, 18–40, 29–42). Cardiac blood sampling was performed under general anesthesia with isoflurane, followed by euthanization by cervical dislocation. For human endpoints, euthanasia by central destruction is performed before the animal becomes unconscious or unresponsive to external stimuli, when it loses more than 20% of its body weight, or when it has severe difficulty walking.

### Animals

Female and male BALB/c mice at 6 to 9 weeks of age were purchased from Clea Japan (Tokyo, Japan). All animals used in this study were maintained under specific-pathogen-free (SPF) conditions in the National Research Center for Protozoan Diseases at Obihiro University of Agriculture and Veterinary Medicine, Obihiro, Japan. The animals used in the current study were treated according to the Guiding Principles for the Care and Use of Research Animals published by the Obihiro University of Agriculture and Veterinary Medicine.

### Plasmid construction

**Figure 1:**
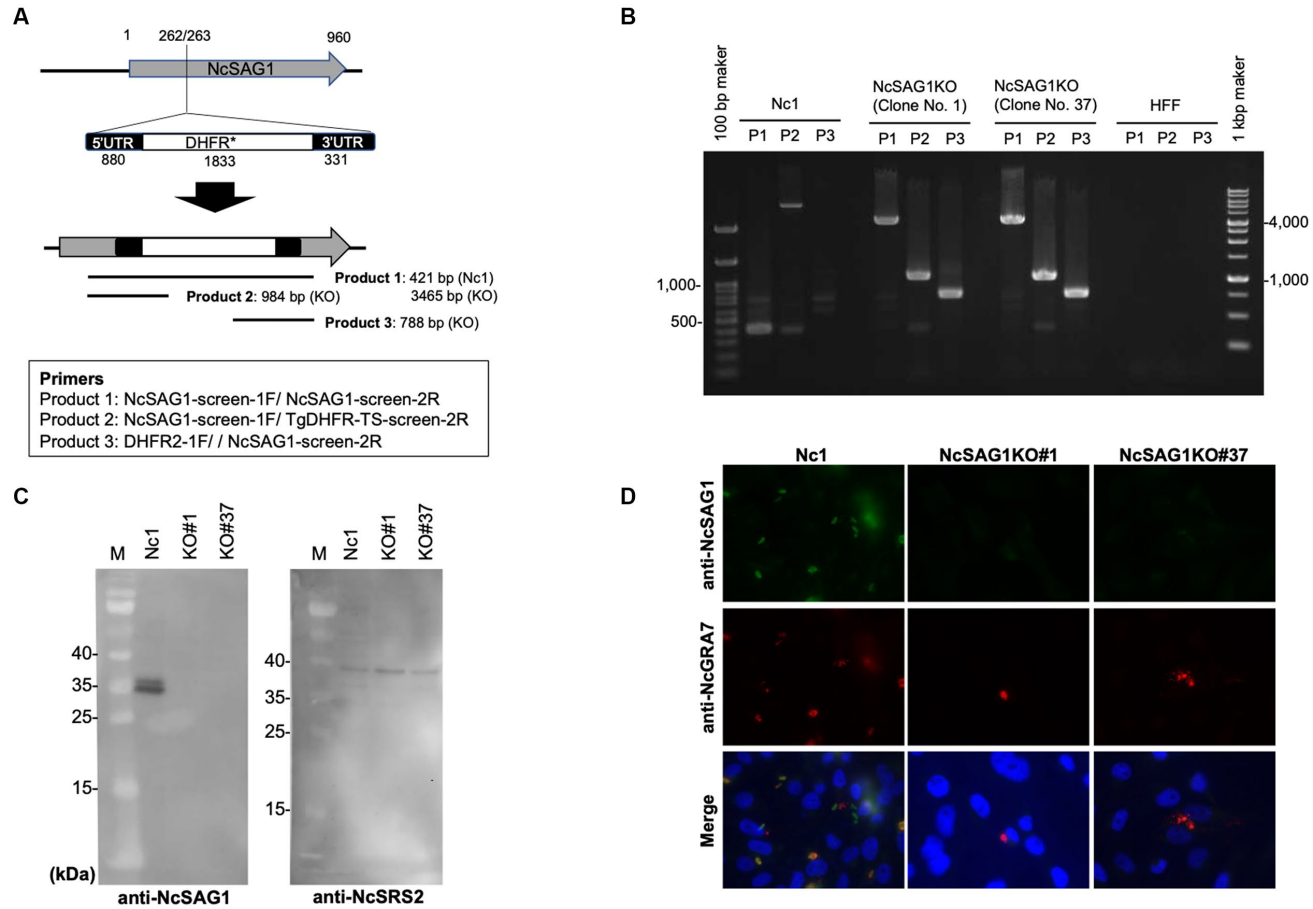
Characterization of NcSAG1-deficient parasites (NcSAG1KO) using PCR, western blotting and IFAT. **(A)** CRISPR/CAS9-mediated disruption of the *NcSAG1* loci. Schematic representation of the CRISPR/CAS9 strategy used to inactivate the target genes by inserting the pyrimethamine-resistance DHFR cassette (DHFR*). Transfection of the CRISPR plasmid targeting the *NcSAG1* gene, together with an amplicon containing the DHFR*-expressing cassette flanked by regions homologous to the target gene, was used to disrupt the corresponding target gene by insertion. Diagnostic PCR demonstrated homologous integration and gene disruption in a representative clone (KO) compared with the parental line Nc1. Product 1 is the fragment amplified in wild-type cells that was lost during the insertion of DHFR*, and the larger fragment was created by the insertion of DHFR* (2.9 kb). Product 2 and Product 3 provide evidence of homologous integration because the products were amplified between the DHFR* gene and regions in the NcSAG1 locus that lie outside the targeted amplicon. Primer set for Product 1 (421 b in Nc1 and 3,465 b in NcSAG1KO): NcSAG1-screen-1F and NcSAG1-screen-2R. Primer set for Product 2 (no amplicon in Nc1 and 984 b in *NcSAG1*KO): NcSAG1-screen-1F and TgDHFR-TS-screen-2R. Primer set for Product 3 (no amplicon in Nc1 and 788 b in NcSAG1KO): DHFR2-1F and NcSAG1-screen-2R. **(B)** PCR results demonstrated the target insertion of the DHFR* cassette into *NcSAG1* gene disruption in NcSAG1KO clones (#1 and #37) compared with the parental line Nc-1. **(C)** western blotting of the parental strain Nc-1 and NcSAG1KO#1 and NcSAG1KO#37. Anti-NcSAG1 monoclonal antibody detected two protein bands (31.4 and 36.1 kDa) in Nc-1 lysate but not in NcSAG1KO clones (left panel). The anti-NcSRS2 monoclonal antibody detected a specific band (40.7 kDa) in all samples. M, molecular mass marker. Each figure panel represents a photo taken from the same blot, including the marker. **(D)** IFAT analysis of Vero cells infected with Nc-1, NcSAG1KO#1 and NcSAG1KO#37 at 24 h postinfection. Cells were fixed and stained with mouse anti-NcSAG1 monoclonal antibody (green), rabbit anti-NcGRA7 (red), and Hoechst dye (blue).

CRISPR plasmids targeting between nucleotides (nt) 262 and 263 in the NcSAG1 gene were constructed for the insertion of the pyrimethamine resistance dihydrofolate reductase (DHFR*) cassette (Figure 1A). Briefly, a single guide RNA (sgRNA) primer was designed using the EuPaGDT website.^1^ A plasmid expressing the CAS9 enzyme and sgRNA targeting the uracil phosphoribosyl transferase (UPRT) gene of *T. gondii* (pSAG1::CAS9U6::sgUPRT) were obtained from Addgene (Cambridge, MA, United States). The Q5® Site-Directed Mutagenesis Kit (New England Biolabs, Ipswich, MA, USA) was used for the generation of the CRISPR/CAS9 plasmid (pSAG1::CAS9-U6::sgNcSAG1) from the common plasmid (pSAG1::CAS9-U6::sgUPRT) by changing the UPRT-targeting sgRNA to NcSAG1-specific sgRNA using the NcSAG1_246-gRNA primer (5′-AAA CAG GAC CGT CTG CCC GGC GG-3′) and the common primer for the CRISPR/CAS9 plasmid targeting *Neospora* genes (5’-AAC TTG ACA TCC CCA TTT AC-3′).

### Generation of NcSAG1 knockout parasites

An amplicon containing the homologous regions surrounding the DHFR* cassette was prepared by PCR amplification using specific primers (DHFR-NcSAG1_246_1F; 5′-TAT CCA ACA AAC AGG ACC GTC TGC CAA GCT TCG CCA GGC TGT AAA-3′, DHFR-NcSAG1_246_2R and 5′-TTT GAC CTC CGG ACT CCG CCG GGA ATT CAT CCT GCA AGT GCA TAG-3′). Approximately 50 μg of the DNA of the previously constructed plasmid (pSAG1::CAS9-U6::sgNcSAG1) with 5 μg of DHFR cassette was used for transfection of wild-type parasites (Nc-1) by electroporation for disruption of the NcSAG1 gene. Then, the transfected parasites were grown in medium containing pyrimethamine antibiotic (1 μM) for two weeks for selection of stably resistant clones. Stably resistant clones were subsequently screened with PCR using specific primers (NcSAG1-screen-1F; 5′-GAA CCA CAT CAC GCT CAA GTG-3′ and NcSAG1-screen-2R; 5’-CGT GCC GCA AAC GAG GGT CAT-3′, NcSAG1-screen-1F and TgDHFR-TS-screen-2R; 5’-CAG ACA CAC CGG TTT CTG CAT-3′, or DHFR2-1F; 5’-CCA TTG TGA ACA TCC TCA AC-3′ and NcSAG1-screen-2R) to ensure the correct integration of DHFR* into the target gene locus (Figures 1A,B). Further analyses using western blot and indirect fluorescent antibody test (IFAT) were applied to confirm the loss of the NcSAG1 gene in the clones (NcSAG1KO).

### Parasites and cell cultures

*N. caninum* (Nc-1 strain) and NcSAG1KO were propagated in Vero cells cultured in Eagle minimum essential medium (Sigma, St. Louis, MO) supplemented with 8% heat-inactivated FBS. For the purification of tachyzoites, parasites and host cell debris were harvested after washing with cold phosphate-buffered saline (PBS) and resuspending the final pellet in cold PBS followed by passing through a 27-gage needle and a 5.0-μm-pore size filter (Millipore, Bedford, MA). The Nc-1 strain and NcSAG1KO strains were cultured up to passage 50 that we have confirmed the stability of the parasites in our lab. The parasites cultures with passage number 20 to 50 were used in this study.

### Western blotting

Lysate antigen from purified tachyzoites of Nc-1 or NcSAG1KO was prepared as described previously (Liao et al., 2005). The obtained extract was filtered through a 0.45 μm low-protein binding Supor® membrane (Pall Life Sciences, Ann Arbor, MI, USA), and the concentration was measured using a bicinchoninic acid (BCA) protein assay kit (Thermo Fisher Scientific, Inc., Rockford, IL, USA). Lysates from purified tachyzoites of *N. caninum* (Nc-1 or NcSAG1KO) were loaded at 1 × 10^6^ tachyzoites/lane. Protein samples were mixed with equal amounts of 2 × SDS gel reducing loading buffer (62.5 mM Tris–HCl pH 6.8, 2% (w/v) SDS, 140 mM 2-mercaptoethanol, 10% (w/v) glycerol and 0.02% (w/v) bromophenol blue) and heated at 95°C for 5 min. Denatured proteins were run on a 12% polyacrylamide gel for band separation. The immunoblotting of samples was performed by transferring protein bands in the gel to a nitrocellulose membrane (Whatman GmbH, Dassel, Germany). The membranes were washed once with PBS and then blocked with PBS containing 3% (w/v) skim milk (PBS-SM) overnight at 4°C. The following day, the membranes were incubated for 1 h at 37°C with diluted mouse monoclonal antibodies (mAbs) against NcSAG1 (2C11) and NcSRS2 (1B8) (1,500 and 1:250, respectively) (Nishikawa et al., 2000) in 3% PBS-SM. Thereafter, the membranes were washed and incubated with horseradish peroxidase-conjugated anti-mouse IgG (1,4,000, Amersham Pharmacia Biotech, Piscataway, NJ, USA) diluted in PBS-SM for 1 h at 37°C. After an additional 3 washes, the protein bands were visualized using ECL™ western blotting detection reagents (GE Healthcare UK Ltd., Buckinghamshire, UK) with a VersaDoc™ imaging system (Nippon Bio-Rad Laboratories, Tokyo, Japan) according to the manufacturer’s recommendations.

### IFAT and *in vitro* growth analysis of *Neospora caninum*

A Vero cell suspension of 1 mL at 5 × 10^4^ cells in MEM supplemented with 8% FBS was plated in each well containing a coverslip in a 12-well plate and incubated at 37°C in a 5% CO_2_ atmosphere for 24 h. The cells were infected with purified tachyzoites at a multiplicity of infection of 1:4 prepared in 1 mL of previously mentioned medium and incubated again at 37°C in an atmosphere of 5% CO_2_ for 24 h, 48 h, and 72 h for assessment of infection, proliferation, and egress rate, respectively. Coverslips were washed once with PBS and then fixed with 3% paraformaldehyde in PBS (v/v) for 30 min at room temperature (RT). After further washing with PBS once, 0.1% Triton X-100 in PBS was added to the cells and kept at RT for 10 min. After washing three times, the coverslips were incubated with 3% bovine serum albumin (BSA) in PBS (BSA-PBS) at RT for 1 h for blocking. As first antibodies, the coverslips were incubated with mouse mAb against NcSAG1 or NcSRS2 (Nishikawa et al., 2000) and rabbit polyclonal anti-NcGRA7 antibodies (Nishikawa et al., 2018) diluted 1:500 in 3% BSA-PBS at RT for 1 h. Then, coverslips were washed three times with PBS at 5 min intervals and incubated with Alexa Fluor 488-conjugated goat anti-mouse IgG and Alexa Fluor 594-conjugated goat anti-rabbit IgG (Sigma) diluted 1:500 in 3% BSA-PBS for 1 h at RT. Nuclear DNA of all samples was marked with Hoechst 33342 (1,1,000 dilution, Thermo Fisher Scientific Inc., MA, USA). Finally, the coverslips were placed on a glass slide containing a fresh drop from Mowiol (Calbiochem, San Diego, CA, USA), and the slides were kept in the dark for at least 2 h before examination using an all-in-one fluorescence microscope (BZ-9000, Keyence, Tokyo, Japan).

At 24 h postinfection, the infection rates were calculated with IFAT as follows: (number of NcSRS2-positive Vero cells/100 randomly selected Vero cells) × 100. To measure parasite replication in Vero cells, the sizes of the parasitophorous vacuoles (PVs) were determined by counting the number of parasites per PV (in a total of 100 randomly selected vacuoles) expressed as a percentage (%) of the total PV at 48 h postinfection based on the NcSRS2 signal measured with IFAT. To measure parasite egress in Vero cells, the percentage of egressed vacuoles was calculated by scoring at least 100 vacuoles as intracellular or egressed vacuoles at 72 h postinfection based on the NcSRS2 signal measured with IFAT.

### *Neospora caninum* infection in mice

To assay the virulence of different parasite strains in mice, nonpregnant female and male BALB/c mice were intraperitoneally inoculated with Nc-1, NcSAG1KO clone #1 or NcSAG1KO clone #37 (1 × 10^6^ tachyzoites/mouse). The mice were monitored for survival rate and body weight for 60 days post infection (dpi). The parasite loads in the brain tissue were measured from succumbed or sacrificed mice. For pathological analysis, nonpregnant female and male BALB/c mice were intraperitoneally inoculated with Nc1 and NcSAG1KO clone #1 (1×10^6^ tachyzoites/mouse). At 28 days post infection, surviving mice were anesthetized and sacrificed by cervical dislocation. The brain was fixed for 24 h in 10% formalin neutral buffer solution.

Regarding the pregnant mouse model, female BALB/c mice (9 weeks old) were housed with male mice at night (one female with one male per cage) and checked in the early morning for the presence of seminal plugs in the vagina. The first day on which a plug was noticed was designated Day 0 of gestation for each individual. All mice were challenged with 1 × 10^5^ tachyzoites of Nc-1 or NcSAG1KO (clone #1) at 3 or 8 days of gestation. Offspring survival rates were observed daily until 30 days after birth. The brain and uterus of all dams and the brain of some died and all surviving offspring at 30 days after birth (corresponding to 42–46 days after infection) were aseptically collected to determine the parasite burden.

### DNA isolation and real-time PCR analysis of *Neospora caninum* distribution

Genomic DNA was extracted from the brain and uterus from tested mice as described previously (Nishikawa et al., 2018). Briefly, each tissue or organ was added to 10 volumes of extraction buffer (0.1 M Tris–HCl [pH 9.0], 1% SDS, 0.1 M NaCl, 1 mM EDTA) and 100 μg/mL proteinase K at 55°C. The DNA was purified with phenol–chloroform extraction and ethanol precipitation. The parasite DNA was then amplified with primers specific to the *N. caninum* Nc5 gene (5’-ACT GGA GGC ACG CTG AAC AC-3′ and 5′- AAC AAT GCT TCG CAA GAG GAA-3′). Amplification, data acquisition, and data analysis were performed in the ABI Prism 7900HT sequence detection system (Applied Biosystems), and the cycle threshold values (CT) were calculated. A standard curve was constructed using 10-fold serial dilutions of *N. caninum* DNA extracted from 10^5^ parasites; thus, the curve ranged from 10 to 100,000 parasites. The parasite number was calculated from the standard curve.

### Pathological analysis

Formalin-fixed brains were cut coronally and embedded in paraffin wax. Three-micrometer-thick sections were stained with hematoxylin and eosin (HE). To evaluate the severity of the pathological lesions in coronal sections from the olfactory bulb to the midbrain, the lesions were scored as follows: 0, no lesions; 1, minimal lesion, characterized by localized perivascular cuffing with a single layer of mononuclear cells; 2, mild lesion, characterized by perivascular cuffing with multiple layers of mononuclear cells and slight parenchymal infiltration; 3, moderate lesions, characterized by multifocal perivascular cuffing and granulomas; and 4, severe lesions, characterized by multifocal perivascular cuffing and granulomas with focal necrosis. The pathological lesions representing the grading criteria are shown in Supplementary Figure S1.

### Statistical analysis

All statistical analyses were performed with GraphPad Prism version 8 (GraphPad Software Inc., La Jolla, CA, USA). Infection, egress rates of the parasite and pathological scores were compared with t tests or Mann–Whitney tests. PV size was analyzed with two-way analysis of variance (ANOVA) followed by the Tukey–Kramer test. The significance of the differences in survival was analyzed with a chi-square test or a log-rank test. The *Neospora*-PCR positive rates were analyzed with a chi-square test. The parasite burden was compared with one-way ANOVA followed by the Tukey–Kramer test, t test or Mann–Whitney test. The levels of statistical significance are presented with asterisks or letters and are defined in each figure legend. A *p* value <0.05 was estimated as statistically significant.

## Results

### Characterization of NcSAG1KO parasites by PCR, western blotting and IFAT

Successful generation of the NcSAG1KO parasites was confirmed by PCR to detect the insertion of the DHFR* cassette into the targeted site. Two clones from NcSAG1KO parasites were selected for further characterization and analyses (NcSAG1KO#1 and NcSAG1KO#37). The amplification of the target gene was negative, and the insertion of the DHFR* cassette into the NcSAG1 gene was confirmed in deficient lines (Figures 1A,B). The absence of target gene expression was also confirmed by western blotting (Figure 1C). Anti-NcSAG1 monoclonal antibody detected 31.4 and 36.1 kDa proteins in the Nc-1 strain but not in the NcSAG1KO clones #1 and #37, while anti-NcSRS2 monoclonal antibody could detect a protein in both Nc-1 and KO clones. Moreover, we confirmed the loss of NcSAG1 expression by IFAT (Figure 1D). All parasite lines were confirmed by finding a specific reaction against NcGRA7, while a specific signal of NcSAG1 was detected only in the Nc-1 parental strain.

### *In vitro* growth kinetic assays

**Figure 2:**
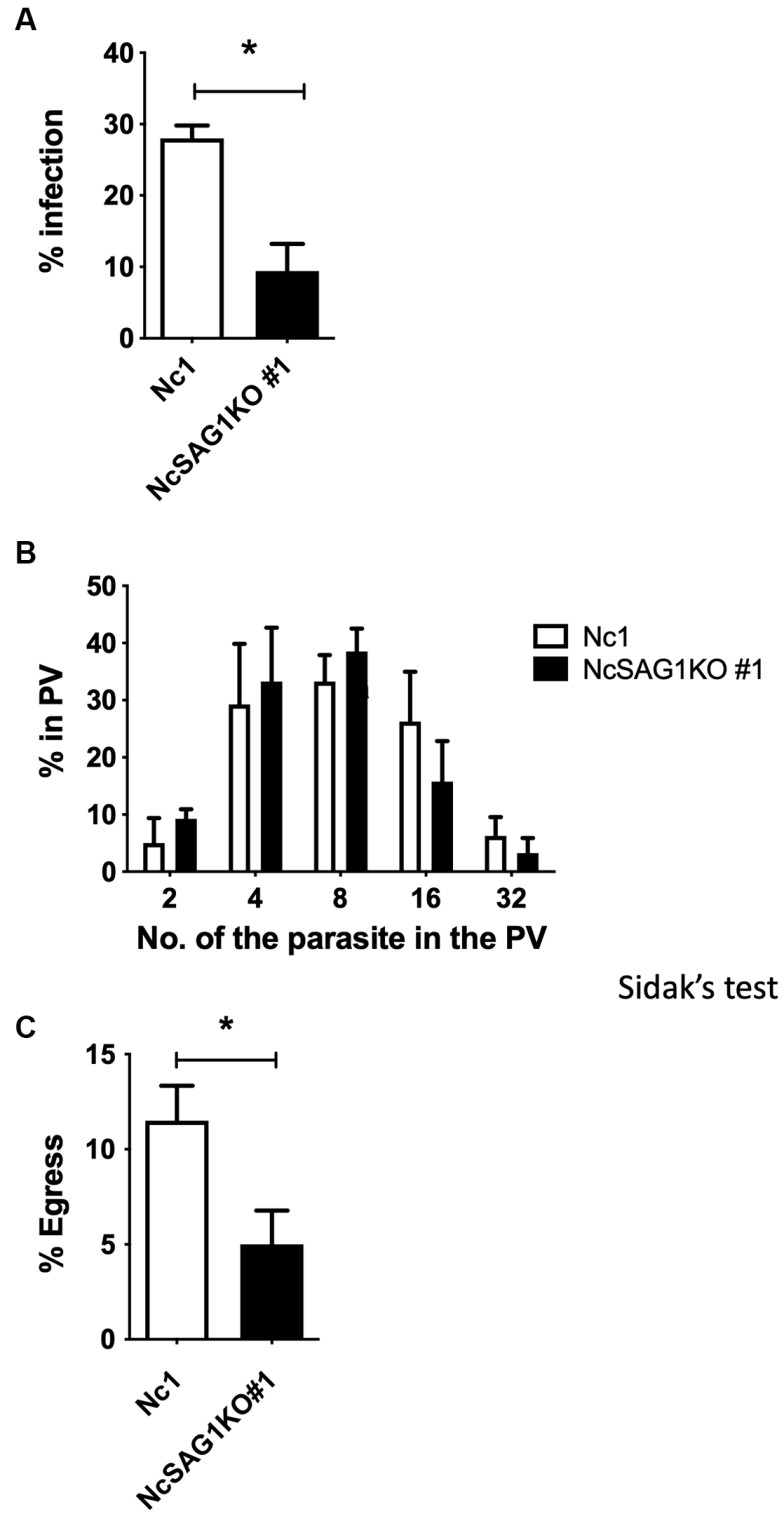
Infection rate, growth, and egress assay of the different parasite lines, Nc-1 and NcSAG1KO#1. **(A)** Infection rates of the parasite lines in Vero cells at 24 h postinfection. **(B)** Intracellular replication assay of the parasite lines in Vero cells at 48 h postinfection. **(C)** Egress rates of the different parasite lines in Vero cells at 72 h postinfection. Each bar represents the mean ± the standard deviation (*n* = 4) for all groups. *, statistically significant differences relative to the value for Nc-1, according to t test **(A,C)** (*p* < 0.05). The parasite number in parasitophorous vacuoles (PV) was analyzed with two-way ANOVA and a Tukey–Kramer *post hoc* analysis, but no significant difference was observed **(B)**.

A statistically significant difference in the infection rates of the NcSAG1KO parasite (clone #1) compared with those of its parental Nc-1 strain in Vero cells at 24 hpi was detected (Figure 2A). Moreover, we measured the numbers of parasites in the PV at 48 hpi between the NcSAG1KO parasites (clone #1) and Nc-1 parasites (Figure 2B), but there was no significant difference. The egress rate of parasites at 72 h postinfection was also measured. The deletion of the NcSAG1 gene significantly impaired the egress of the parasite (Figure 2C). Another clone #37 of NcSAG1KO also showed similar results of *in vitro* growth kinetics compared with the NcSAG1KO parasite (clone #1) (Supplementary Figure S2).

### Virulence assay in mice

**Figure 3:**
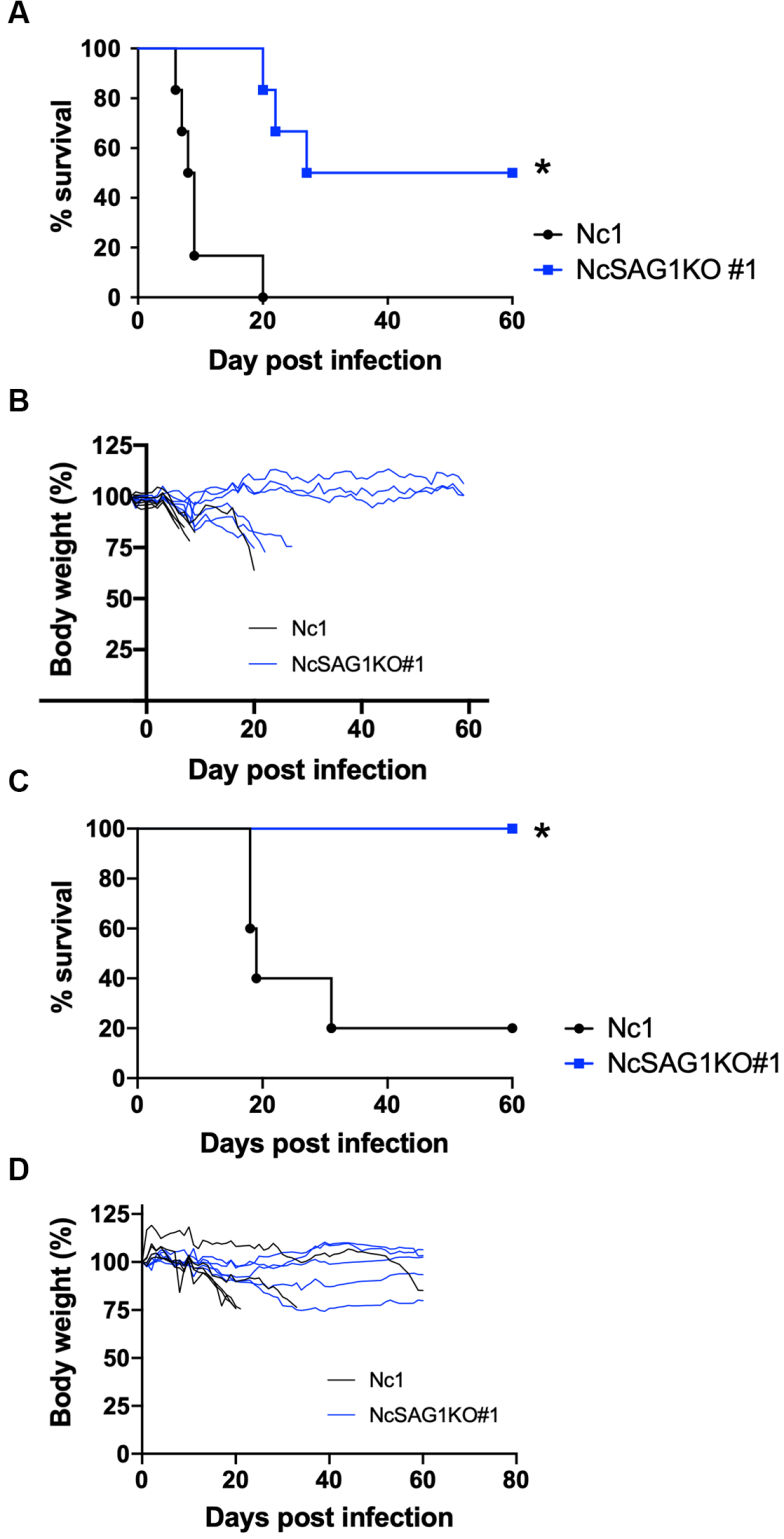
Virulence assay in a nonpregnant mouse model. BALB/c mice were infected intraperitoneally with a lethal dose (1 × 10^6^) of *N. caninum* tachyzoites of the parental strain Nc-1 and NcSAG1KO#1. Mouse survival and changes in body weight were calculated for 60 days post infection (dpi). Survival curves **(A)** and body-weight changes **(B)** of female BALB/c mice were measured. Survival rates (n = 6 per group): Nc1, 0/6, 0%; NcSAG1KO#1, 3/6, 50.0%. The significance of the differences in survival was analyzed with a log-rank test (*, *p* < 0.05). Survival curves **(C)** and body-weight changes **(D)** of male BALB/c mice were measured. Survival rates (*n* = 5 per group): Nc1, 1/5, 20%; NcSAG1KO#1, 5/5, 100%. The significance of the differences in survival was analyzed with a log-rank test (*, *p* < 0.05).

The virulence of the Nc-1 and NcSAG1KO parasites was analyzed using nonpregnant female BALB/c mice (Figures 3A,B). The mice infected with the NcSAG1KO parasite (clone #1) showed higher survival rates and lower body weight loss in relation to the parental strain Nc-1. As shown in Supplementary Figure S3, another clone #37 of NcSAG1KO also showed lower virulence to nonpregnant female BALB/c mice. Next, we examined the susceptibility of male BALB/c mice to infection (Figures 3C,D). Similar to the case of nonpregnant female BALB/c mice, significantly higher survival rates and little body weight loss were confirmed in the infection with NcSAG1KO parasite (clone #1) compared with those with Nc1.

### Pathological analysis

**Figure 4:**
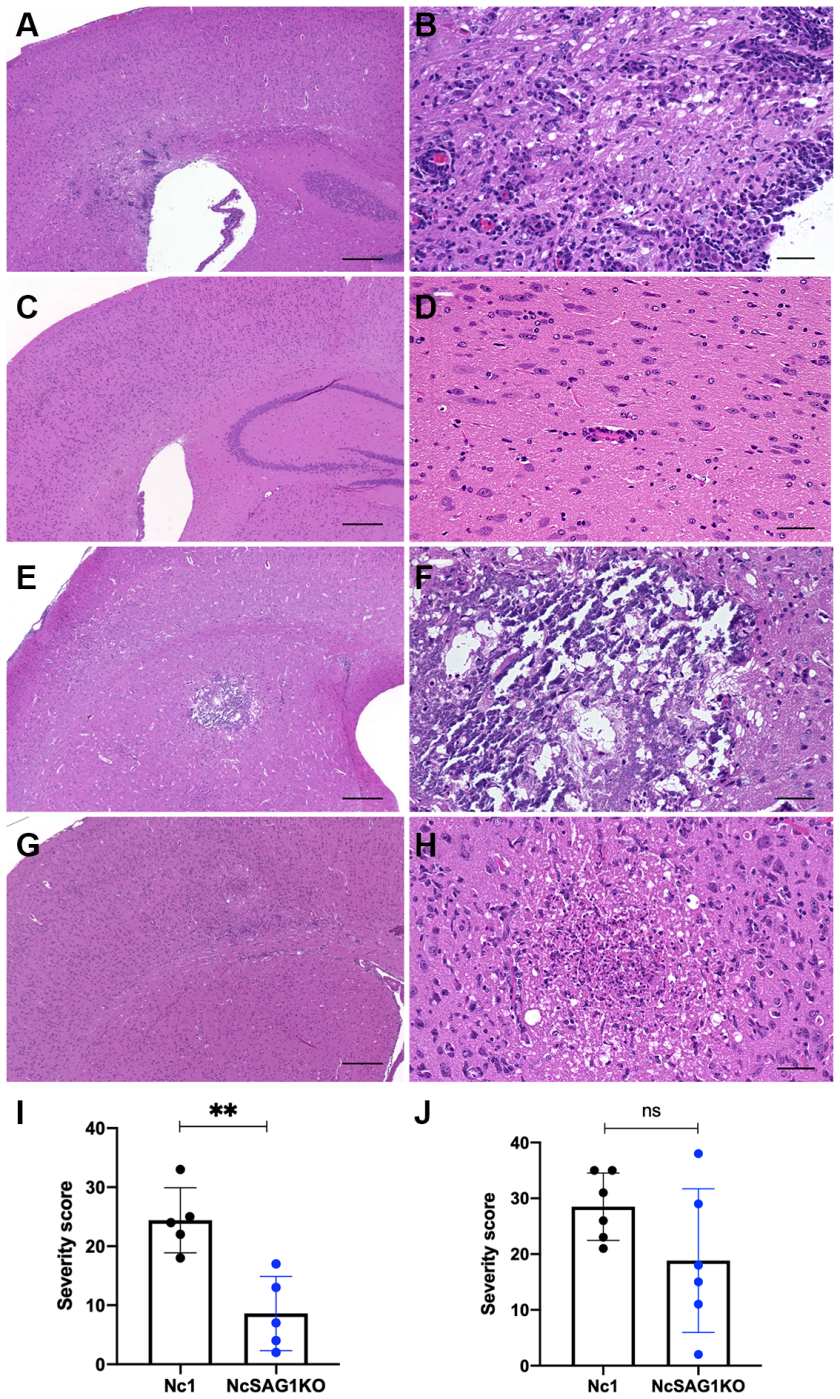
Inflammatory and necrotic lesions in male and female mice infected with Nc1 or NcSAG1KO. **(A)** Inflammatory and necrotic lesions in male mice infected with Nc1. **(B)** High magnification of the inflammatory lesion in **(A)**. **(C)** Inflammatory and necrotic lesions in male mice infected with NcSAG1KO#1. **(D)** High magnification of the inflammatory lesion in **(C)**. **(E)** Inflammatory and necrotic lesions in female mice infected with Nc1. **(F)** High magnification of the inflammatory lesion in **(E)**. **(G)** Inflammatory and necrotic lesions in female mice infected with NcSAG1KO. **(H)** High magnification of the inflammatory lesion in (G). Scale bar = 300 μm **(A,C,E,G)**; 50 μm **(B,D,F,H)**. **(I)** The severity score of the pathological lesions in male mice infected with Nc1 or NcSAG1KO. **(J)** The severity score of the pathological lesions in female mice infected with Nc1 or NcSAG1KO. The significance of the differences in the severity score was analyzed with the Mann–Whitney test (*, *p* < 0.05).

Multifocal inflammatory and necrotic lesions and meningitis were observed in the infected mice (Figures 4A–H). The lesions were mainly distributed in the cerebrum and brainstem. Minimal to severe inflammatory and necrotic lesions characterized by perivascular cuffing were observed in both the cerebral cortex and brainstem. In moderate inflammatory lesions, there is perivascular and parenchymal infiltration of mononuclear cells, including lymphocytes, macrophages and epithelioid cells. Some necrotic lesions were accompanied by infiltration of foamy macrophages and calcification. Statistical analysis revealed that pathological lesions were significantly more severe in male mice infected with Nc1 than in those infected with NcSAG1KO (Figure 4I). Although a similar trend was observed in female mice, it did not reach statistical significance (Figure 4J).

### The effects of NcSAG1 deletion on vertical transmission of *Neospora caninum*

**Figure 5:**
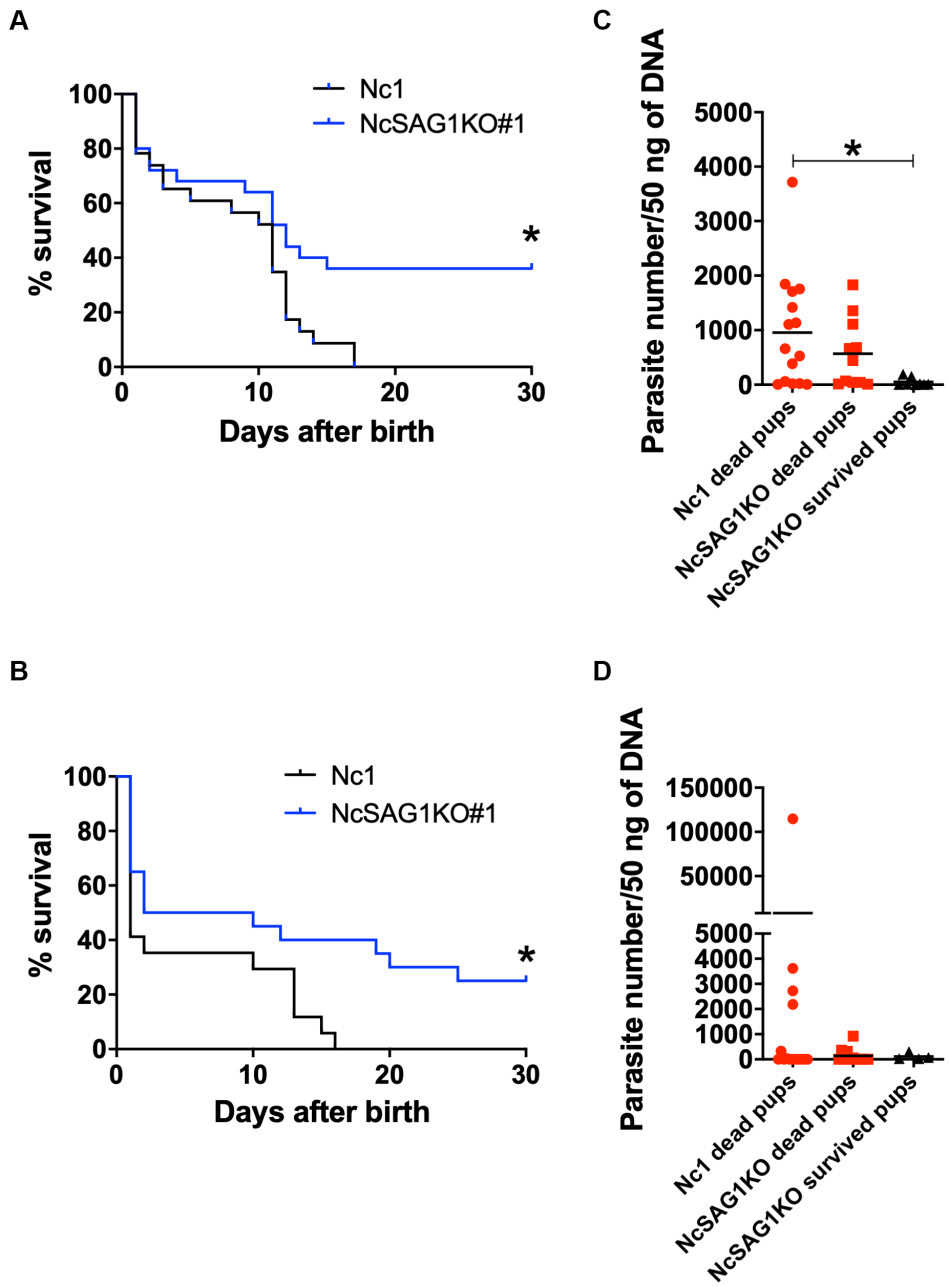
Survival rates and parasite burden in the brains of offspring. Female mice were infected with a nonlethal dose of Nc-1 and NcSAG1-deficient parasites (NcSAG1KO#1) at 8 days after confirmation of vaginal plugs. Offspring from dams were monitored daily to calculate the survival rate and collect brains from the offspring. The survival rates of offspring were calculated until 30 days after birth for trial-1 **(A)** and trial-2 **(B)**. Survival curves were generated with the Kaplan–Meier method. According to the log-rank test, the difference was significant between the Nc-1 and NcSAG1KO groups in both experiments (* *p* < 0.05). The parasite numbers in the brains of the deceased and surviving offspring at 30 days after birth are indicated for trial-1 **(C)** and trial-2 **(D)**. Some brains of dead offspring could not be collected because of cannibalistic behavior by dams. *, statistically significant differences were observed with one-way ANOVA followed by the Tukey–Kramer test. The red-colored symbols indicate that the samples were collected from dead mice. Pregnancy results and survival rates of offspring (30 days postpartum) are also shown in Table 1.

Because the two clones of NcSAG1KO showed similar biological activities, we assayed the role of NcSAG1 using NcSAG1KO (clone #1) in a pregnant BALB/c mouse model (Figure 5). Two independent experiments were performed to elucidate the role of NcSAG1 in infection during pregnancy. The parasite number in the brains of NcSAG1KO-infected dams was significantly lower than that of Nc-1-infected dams, while there was no significant difference in the parasite number in the uterus (Supplementary Figure S4). The fertility rate, birth rate and mean number of offspring per litter of the infected mice showed no significant difference from those of noninfected mice (Table 1). We did not observe abnormal pregnancy, such as no birth, still birth or delayed birth, in either infected mouse group (Table 1). The survival rates of offspring from uninfected mothers were calculated up to 30 days after birth, yielding a value of 77.8% (Table 1). Although the survival rates of offspring in both infected mouse groups decreased compared with those in the noninfected mouse group (Table 1), a significantly higher survival of offspring from NcSAG1KO-infected dams was observed compared with those from Nc-1-infected dams (Table 1 and Figures 5A,C). Additionally, there were no surviving offspring from Nc-1-infected dams, and the infection rate was 61.3% based on the parasite DNA in the brain. In the NcSAG1KO-infected group, the parasite number in the brain from the surviving offspring showed a lower tendency than that from the dead mice (Figures 4B,D). The infection rate of the offspring from the NcSAG1KO-infected dams was 48.6%, while there was no significant difference in the infection rates between the Nc-1-infected and NcSAG1KO-infected groups (Table 1).

**Table 1:** Pregnancy results and survival rates of offspring (30 days postpartum) following N. caninum infection.

				Abnormal pregnancy							
Groups	Trial	Number of litters (no. used mice), fertility rate %	Birth rate (%)	No birth	Still birth	Delayed birth	Mean number of offspring/litter (SD)	No. of surviving offspring/no. of offspring in each litter	Total no. of surviving offspring /total no. of offspring (survival rate %)	Number of PCR po-sitive offspring in each litter/number of analyzed samples in each litter**	Total Number of PCR positive offspring / total number of analyzed samples (infection rate, %)
Nc1-infected	1	3 (6) 50%	3/3 (100%)	0/3 (0%)	0/3 (0%)	0/3 (0%)	8 (1)	0/8, 0/7, 0/9	0/24 (0%)	4/4, 4/4, 5/7	13/15 (86.7%)
	2	2 (3) 66.6%	2/2 (100%)	0/2 (0%)	0/2 (0%)	0/2 (0%)	9 (NA)	0/10, 0/8	0/18 (0%)	3/9, 3/7	6/16 (37.5%)
	Total	5 (9) 55.6%	5/5 (100%)	0/5 (0%)	0/5 (0%)	0/5 (0%)	8.4 (1.1)		0/42 (0%)#, $		19/31 (61.3%)#
NcSAG1KO-infected	1	3 (6) 50%	3/3 (100%)	0/3 (0%)	0/3 (0%)	0/3 (0%)	8 (2)	0/10*, 3/8, 5/6	8/24 (33.3%)	5/7*, 6/7, 0/5	11/19 (57.93%)
	2	3 (3) 100%	3/3 (100%)	0/3 (0%)	0/3 (0%)	0/3 (0%)	8.3 (2.1)	0/10, 1/9, 4/6	5/25 (20%)	1/6, 3/6, 2/4	6/16 (37.5%)
	total	6 (9) 66.7%	6/6 (100%)	0/6 (0%)	0/6 (0%)	0/6 (0%)	8.2 (1.8)		13/49 (26.5%)#		17/35 (48.6%)#
Non-infected		3 (5) 60%	3/3 (100%)	0/3 (0%)	0/3 (0%)	0/3 (0%)	6 (2.6)	2/4, 8/9, 4/5	14/18 (77.8%)	0/4, 0/9, 0/5	0/18 (0%)

*All pups died within 10 days after birth. The mother was weak and lost a lot of its body weight (34%) then died after 15 days of delivery.

** Some brains were not collected due to cannibalistic behavior of dam.

Significanr diferrences were analyzed with a χ2 test (# *p* < 0.05 vs Non-infected, $ *p* < 0.05 vs NcSAG1KO-infected).

## Discussion

Although *N. caninum* infects canines as a definitive host and negatively affects animal welfare, most economic losses are attributable to induced abortion in cattle. In our previous study, we found that anti-NcSAG1 antibodies are remarkably elevated in the case of aborted cows compared to those in infected but not aborted cows (Abdelbaky et al., 2020). This observation was also preceded by some other reports that recorded high antibody levels of NcSAG1 in aborted cattle (Huang et al., 2007; Hiasa et al., 2012; Takashima et al., 2013). Therefore, in the current study, the role of NcSAG1 was investigated in terms of the virulence, abortion and vertical transmission of *N. caninum* through the generation of the NcSAG1KO parasite.

The results of the growth kinetics of the parasite showed a significant decrease in the infection rate and the egress rate of the NcSAG1KO parasite compared with those of the parental parasite. Previously, we showed that a monoclonal antibody against NcSAG1 inhibited the invasion of *N. caninum* into host cells (Nishikawa et al., 2000). Therefore, we could provide evidence that NcSAG1 plays a crucial role in the parasite invasion step. Unexpectedly, NcSAG1 may have a role in parasite egress, but it is necessary to clarify the detailed function. *In vivo* results using nonpregnant female and male mice revealed the efficient role of NcSAG1 in the virulence of the parasite, as indicated by the markedly higher survival rate and lower body weight change in NcSAG1KO-infected mice than in Nc-1-infected animals. Thus, the biological function of invasion and egress will contribute to parasite virulence in host animals. These results are consistent with those obtained by our previous study using the NcGRA7KO parasite (Nishikawa et al., 2018); the lack of the NcGRA7 gene reduced the egress rate of the parasite *in vitro* and the parasite burden *in vivo* and thus increased the survival of the infected mice.

An investigation into the possible role of the NcSAG1 gene in the abortion and/or vertical transmission of *N. caninum* was conducted in a pregnant mouse model. Infection of pregnant mice was performed at two different time points, 3 and 8 days after confirmation of vaginal plug, representing the early and the mid stages of pregnancy, respectively. However, the results were highly similar in both experiments either in dams or in their offspring (Figure 4 and Supplementary Figure S5). Overall, the survival of offspring is increased due to the lower infection rate in the NcSAG1KO-infected group than in the Nc-1-infected group. Additionally, the burden of NcSAG1KO also decreased in the brains of the dams compared with that of Nc-1. In our experimental conditions, abnormal pregnancy (no birth, still birth and delayed birth), including abortion and fetal absorption, was not observed. These results suggest that NcSAG1 is an essential molecule in the pathogenesis and vertical transmission of neosporosis in pregnant mice.

To further confirm the role of NcSAG1, analysis using *NcSAG1*-complemented parasites is useful. We established a method to generate *NcGRA7*-complemented parasite insertion of the NcGRA7 gene into the *Neospora* uracil phosphoribosyl transferase (NcUPRT) gene (Nishikawa et al., 2018), but we failed to obtain *NcSAG1*-complemented parasites. Although we tried other methods, such as insertion of the NcSAG1 gene into the *Neospora* hypoxanthine-xanthine-guanine phosphoribosyltransferase (HXGPRT) gene (Mineo et al., 2022) or random insertion of the NcSAG1 gene into the *N. caninum* genome (Abe et al., 2015), we could not obtain the *NcSAG1*-complemented parasite. Thus, we confirmed the results using two different clones of NcSAG1KO parasites in this study.

Numerous previous studies have revealed the efficient potential of NcSAG1 in stimulating cellular or humoral immune responses [reviewed thoroughly by Hemphill and Müller (2015); Aguado-Martinez et al. (2017); Sinnott et al. (2017); Fereig and Nishikawa (2020)]. However, the immunomodulatory effect of NcSAG1 is more likely relevant to the cellular than the humoral immune response (Kato et al., 2015; Marin et al., 2017). Some previous studies revealed the importance of Th-2-mediated immunity, especially IL-4, in maintaining pregnancy in mice infected with *N. caninum*, in contrast to IFN-γ (Gao et al., 1996; Kano et al., 2007). However, the effects of NcSAG1 on immune responses are unknown in our study. Our current datasets suggest that parasite invasion and egress steps are involved in the pathogenesis of neosporosis and vertical transmission, but the contribution of host-immune responses should be further examined in future studies.

Surface antigens of tissue-dwelling coccidia including *T. gondii*, *N. caninum* and *Sarcocystis* are involved in the process of attachment and invasion of the parasite to host cells, activating the host immunity and regulating the pathogenesis (Jung et al., 2004). Therefore, it is noteworthy to highlight the marked disparity in SAG1-related sequences (SRS) abundance among the parasites. The genomic sequencing studies of *T. gondii* revealed the existence of a family of 109 SRS genes with two subfamilies, with SAG1 and SAG2A as prototypic members in the Me49 strain (Jung et al., 2004; Wasmuth et al., 2012). On the other hand, putative orthologs with SRS domains of 29 genes were found in the EST data from *N. caninum* and *S. neurona* (Wasmuth et al., 2012), indicating fewer number of the surface antigen compared with *T. gondii*. SAG1 (SRS29B)-deficiency in *T. gondii* do not exhibit marked effects on mouse survival against the infection (Wasmuth et al., 2012) while our study showed NcSAG1KO parasites decreased the virulence in mice. These results suggested that the varied expression of SRS may complement the SAG1 function in the SAG1-deficient *T. gondii*, but NcSAG1-deficiency in *N. caninum* clearly exhibited the significant effects on *in vitro* culture and mouse survival due to the little variety of surface antigens.

Our data suggests that NcSAG1KO decreased the virulence, considering an avirulent strain. Unfortunately, our lab does not have other strains of *N. caninum*, but comparative study using several strains regarding NcSAG1 function will be important for future study. In our preliminary data, immunization with NcSAG1KO protected mice against challenge infection with *N. caninum*. Thus, we are now evaluating possibility of NcSAG1KO as vaccine strain. Exploring the distinctions in the immune response of NcSAG1KO strain is not only beneficial in the present context but also lays the foundation for the development of a future vaccine based on an attenuated strain.

Understanding the mechanism of *Neospora* abortion and the identification of the virulence factors is beneficial for providing a new target for treatment or vaccine development. Vaccine is the best disease control strategy as long as it protects against fetal loss, avoids vertical transmission. Our previous results suggested potential role of NcSAG1 as a virulence factor which contributes to the abortion onset or the vertical transmission of *N. caninum*. Herein, we aimed to unravel the role of NcSAG1 gene as a virulence factor of *N. caninum* parasite and its possible role in the vertical transmission of the parasite through the generation of NcSAG1KO parasite using CRISPR/Cas9 technique in mouse model. Our results suggest that parasite invasion and egress steps are involved in the pathogenesis of neosporosis and vertical transmission, that the deletion of NcSAG1 decreased the virulence of the parasite. Additionally, determining host ligands that physically bind with NcSAG1 is important for understanding how *N. caninum* infects such a wide spectrum of cell types from different hosts. Their ligands could also be target for the parasite invasion, thus reconciling their role as both adhesins and immune targets. Accordingly, our study presented NcSAG1 as a target molecule for vaccine development for neosporosis elimination.

## Data availability statement

The original contributions presented in the study are included in the article/Supplementary material, further inquiries can be directed to the corresponding author.

## Ethics statement

The animal study was approved by Committee on the Ethics of Animal Experiments at the Obihiro University of Agriculture and Veterinary Medicine. The study was conducted in accordance with the local legislation and institutional requirements.

## Author contributions

HA: Data curation, Formal analysis, Investigation, Writing – original draft. MMR: Data curation, Investigation, Writing – review & editing. NS: Data curation, Investigation, Writing – review & editing. YC: Data curation, Investigation, Writing – review & editing. TH: Data curation, Investigation, Writing – review & editing. NU: Data curation, Investigation, Writing – review & editing. YN: Data curation, Investigation, Writing – review & editing, Conceptualization, Formal analysis, Funding acquisition, Methodology, Project administration, Resources, Supervision, Visualization.

## Footnotes

^1^http://grna.ctegd.uga.edu
