# Supplementary material for: Neospora caninum surface antigen 1 is a major determinant of the pathogenesis of neosporosis in nonpregnant and pregnant mice
Source: Front Microbiol. 2024 Jan 8;14:1334447. doi: 10.3389/fmicb.2023.1334447 (PMC10800813; doi:10.3389/fmicb.2023.1334447)
Supplement: Supplementary file 1 [file Data_Sheet_1.PDF]

Supplemental information file

Some parts in this manuscript have been written in doctoral theses of the first author in this manuscript, Dr. Hanan H. Abdelbaky. Her doctoral theses granted by Obihiro University of Agriculture and Veterinary Medicine are made available on the internet through the University Repository.
https://obihiro.repo.nii.ac.jp/records/4699

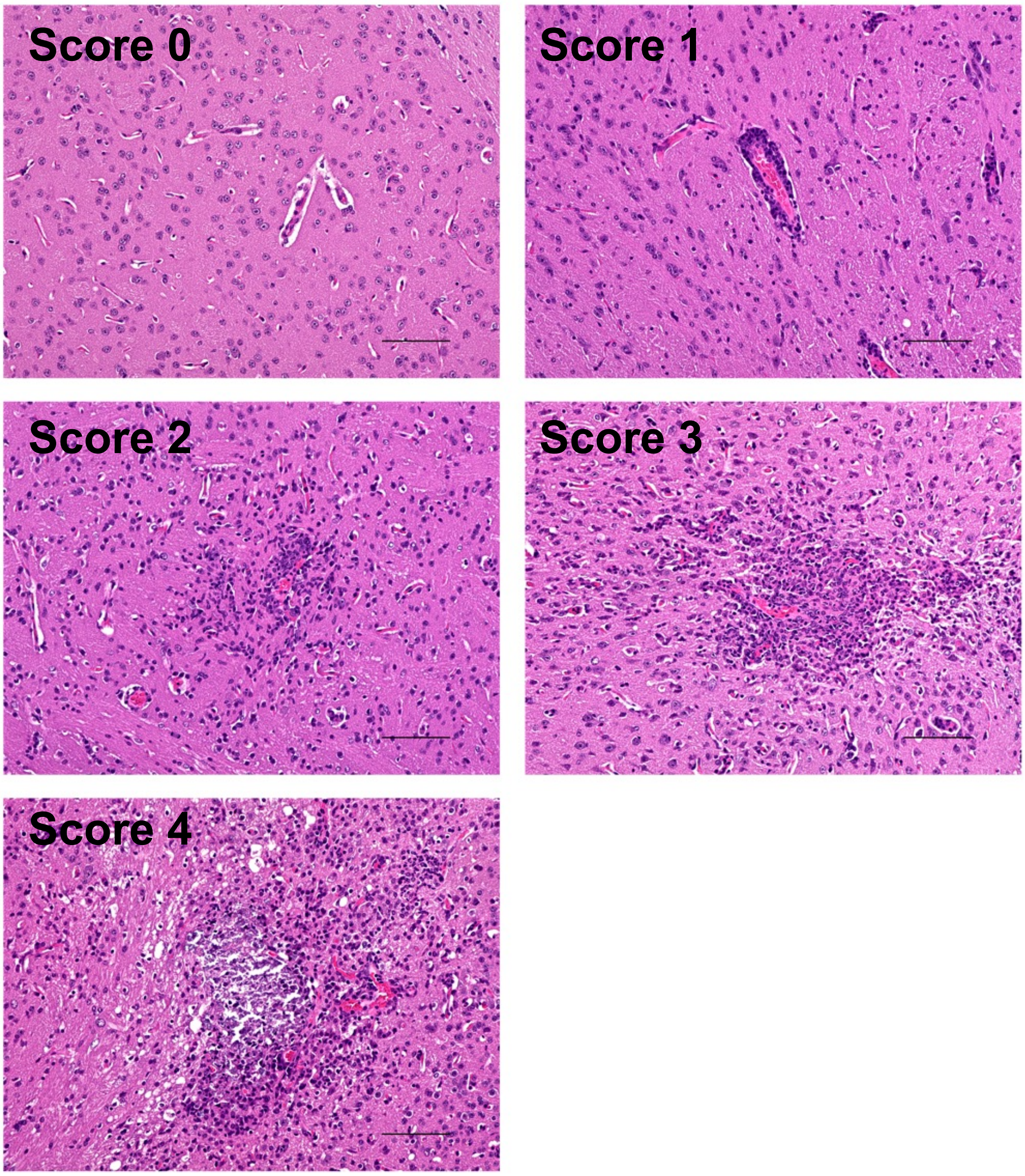

Fig. S1. Examples of grading criteria for histopathological lesions in the brain. Representative examples of histopathological lesions in the brains of mice infected with Nc1 or NcSAG1KO, score 0, no lesions; score 1, minimal lesion, localized perivascular cuffing; score 2, mild lesion, perivascular cuffing with multiple layers of mononuclear cells and slight parenchymal infiltration; score 3, moderate lesion, characterized by multifocal perivascular cuffing and granulomas; score 4, severe lesions, multifocal perivascular cuffing and granulomas with focal necrosis. Scale bar= 100 μm.

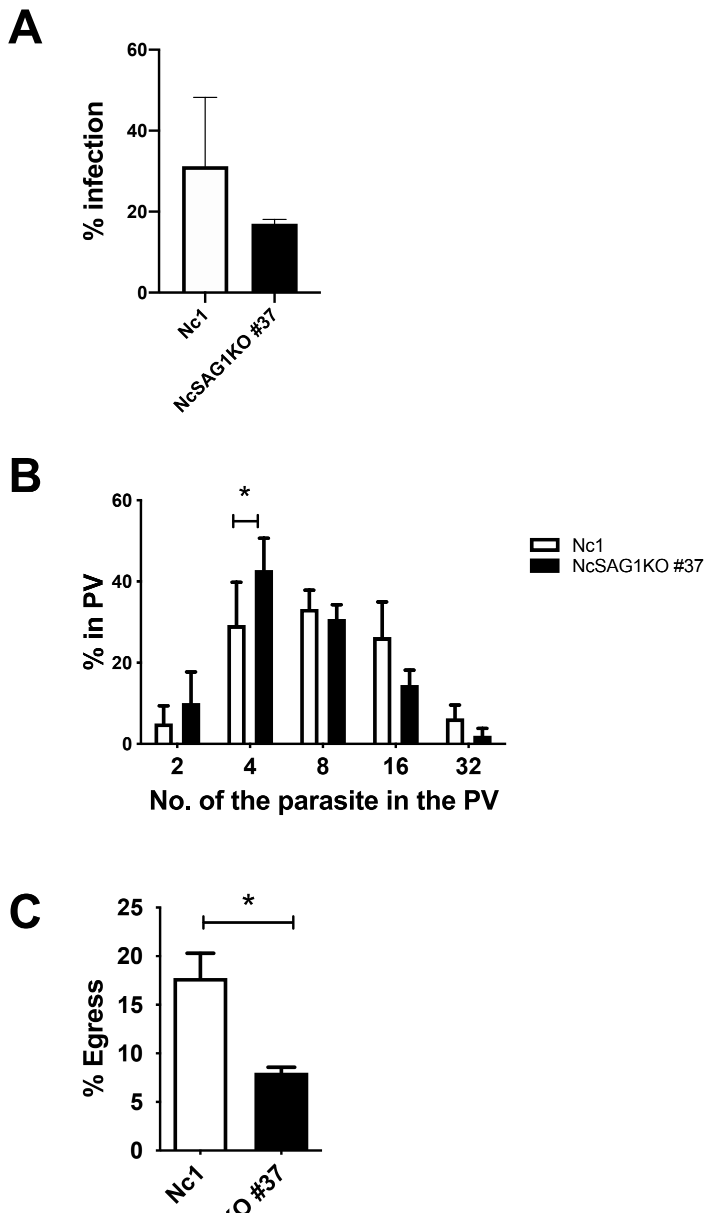

Fig. S2. Infection rate, growth, and egress assay of the NcSAG1KO parasite (clone 37). The data represent the infection rates (A), intracellular replication assay (B) and egress rates (C) of Nc-1 and NcSAG1KO#37 in Vero cells at 24, 48 and 72 h postinfection, respectively. Each bar represents the mean ± the standard deviation (n = 3-4) for all groups. *, statistically significant differences relative to the value for Nc-1, according to Mann–Whitney test (A) or t test (C) ($P < 0.05$). The parasite number in parasitophorous vacuoles (PV) was analyzed with two-way ANOVA and a Tukey–Kramer post hoc analysis (* $P <$ 0.05).

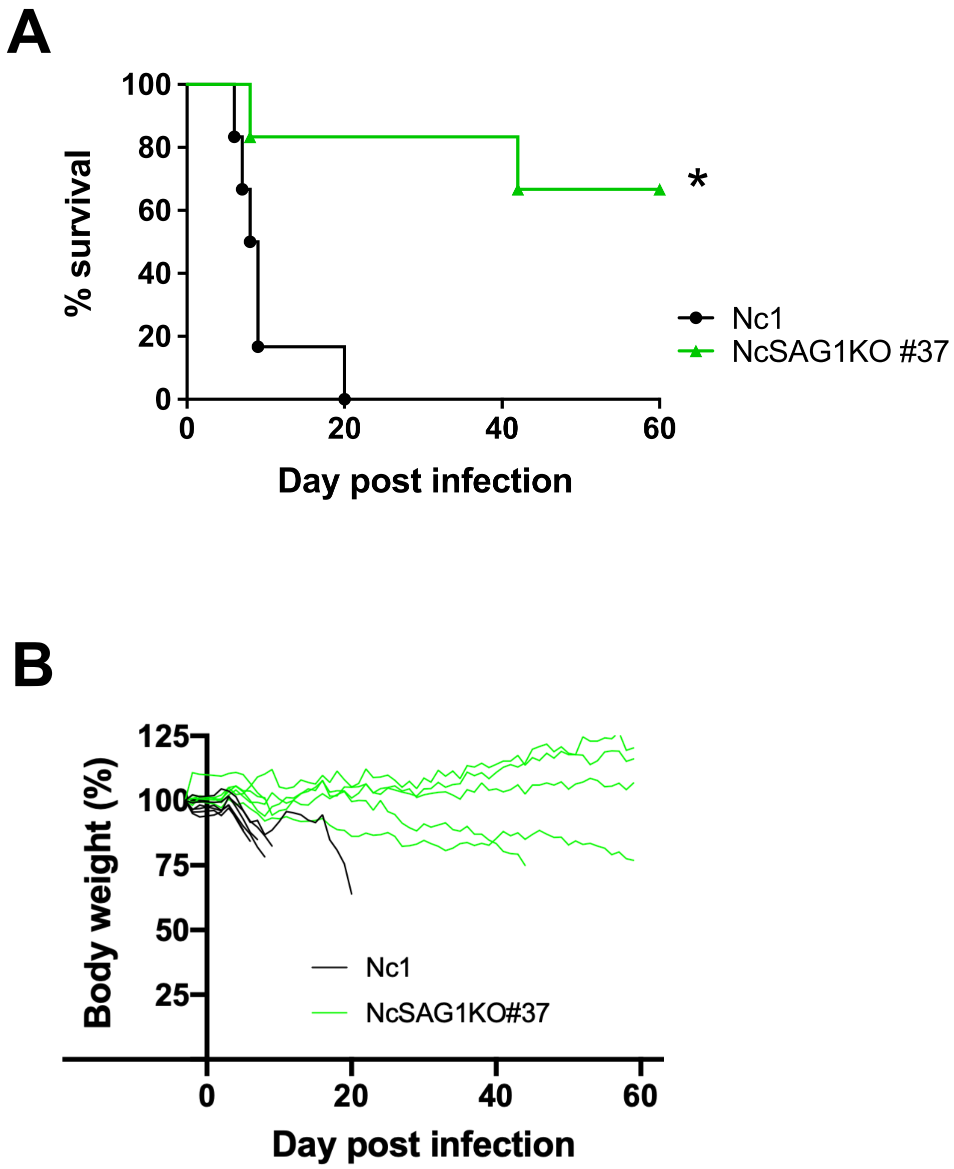

Fig. S3. Virulence assay in a nonpregnant mouse model. BALB/c mice were infected intraperitoneally with a lethal dose (1×10^6) of *N. caninum* tachyzoites of the parental strain Nc-1 and NcSAG1KO#37. Mouse survival and changes in body weight were calculated for 60 days post infection (dpi). Survival curves (A) and body-weight changes (B) of female BALB/c mice were measured. Survival rates (n = 6 per group): Nc1, 0/6, 0%; NcSAG1KO#1, 4/6, 66.7%. The significance of the differences in survival was analyzed with a log-rank test (*, $P < 0.05$).

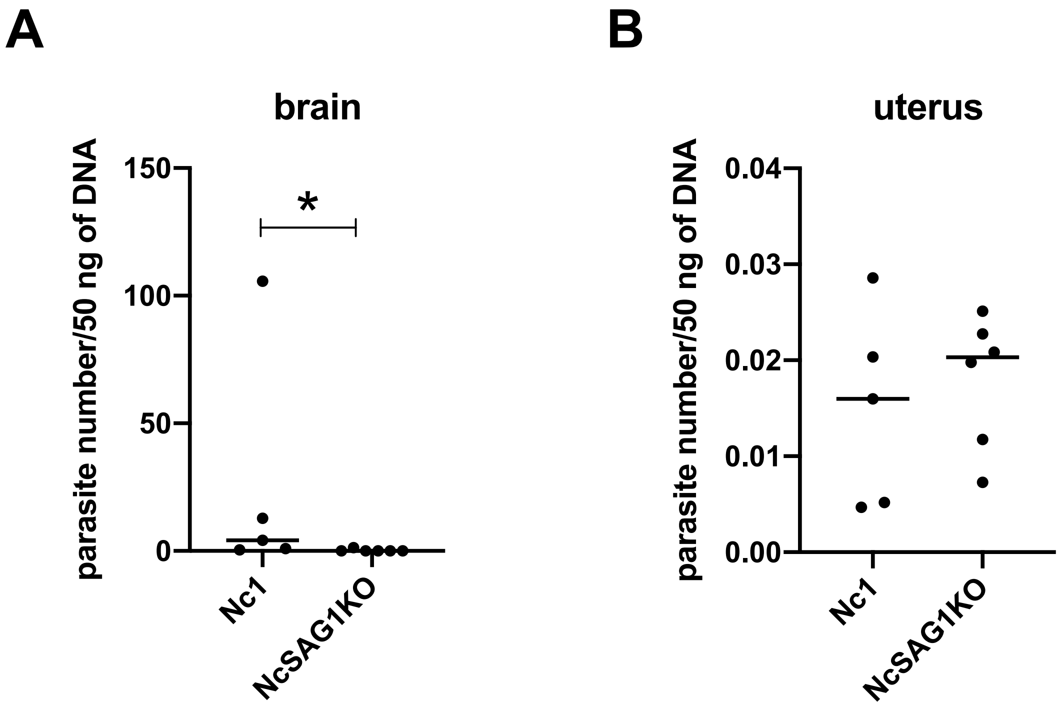

Fig. S4. Parasite burden in the brain and uterus of pregnant dams. In a pregnant mouse model, female BALB/c mice were infected intraperitoneally with a nonlethal dose of Nc-1 or NcSAG1-deficient parasites (NcSAG1KO#1) (1×10^5). Female mice were infected at 8 days after confirmation of a vaginal plug. Parasite burden was measured in the brains (A) and uterus (B) from the dams in two trials (Nc-1-infected, n=5; NcSAG1KO-infected, N=6). *, statistically significant differences according to Mann–Whitney test ($P < 0.05$).

A

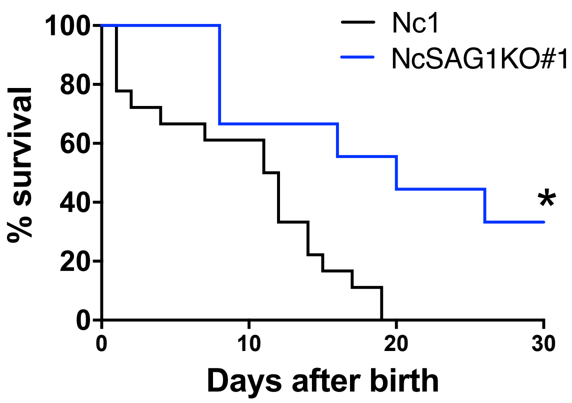

B

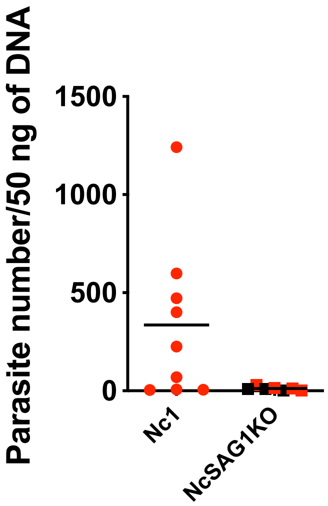

Fig. S5. Survival rates and parasite burden in the brains of offspring. Female mice were infected with a nonlethal dose of Nc-1 (n=3) and NcSAG1-deficient parasites (NcSAG1KO#1) (n=2) at 3 days after confirmation of vaginal plugs. Offspring from dams were monitored daily to calculate the survival rate and collect brains from the offspring. The survival rates of offspring were calculated until 30 days after birth (A). Survival curves were generated with the Kaplan–Meier method. According to the log-rank test, the difference was significant between the Nc-1 and NcSAG1KO groups (* $P < 0.05$). The parasite numbers in the brains of the deceased and surviving offspring at 30 days after birth are indicated (B). Some brains of dead offspring could not be collected because of cannibalistic behavior by dams.
